# Covered SEMS failed to cure airway fistula closed by an amplatzer device

**DOI:** 10.1186/s12890-023-02548-8

**Published:** 2023-07-20

**Authors:** Huibin Lu, Yahua Li, Kewei Ren, Zongming Li, Juanfang Liu, Xuhua Duan, Jianzhuang Ren, Xinwei Han

**Affiliations:** 1 Department of Interventional Radiology, The First Affiliated Hospital of Zhengzhou University, 450052 Zhengzhou, PR China; 2 Interventional Institute of Zhengzhou University, 450052 Zhengzhou, PR China; 3 Interventional Treatment and Clinical Research Center of Henan Province, 450052 Zhengzhou, PR China

**Keywords:** Airway fistula, Stent, Amplatzer device

## Abstract

**Background:**

Airway fistula is a rare but threatening complication associated with high rates of morbidity and mortality. We report the experience of Amplatzer device application in airway fistulae that failed to be cured with a covered self-expandable metallic stent (SEMS).

**Materials and methods:**

Patients who failed occlusion with a covered self-expandable metallic stent and received Amplatzer device placement from Jan 2015 to Jan 2020 were retrospectively enrolled. A total of 14 patients aged 42 to 66 years (55.14 ± 7.87) were enrolled in this study. The primary diseases, types of fistula, types of stents, duration, size of fistula, and follow-up were recorded.

**Results:**

All 14 patients with airway fistula failed to be occluded with a covered metallic stent and received Amplatzer device placement. Among the 14 patients, 6 had BPF, 3 had TEF and 5 had GBF. The average stent time was 141.93 ± 65.83 days. The sizes of the fistulae ranged from 3 to 6 mm. After Amplatzer device placement, the KPS score improved from 62.14 ± 4.26 to 75.71 ± 5.13 (P < 0.05). No procedure-related complications occurred. During the 1-month, 3-month and 6-month follow-ups, all the Amplatzer devices were partially surrounded with granulation. Only 1 patient with BPF failed with Amplatzer device occlusion due to the recurrence of lung cancer.

**Conclusion:**

In conclusion, the application of the Amplatzer device is a safe and effective option in the treatment of airway fistula that failed to be occluded with SEMSs.

**Supplementary Information:**

The online version contains supplementary material available at 10.1186/s12890-023-02548-8.

## Background

Airway fistula is a rare but threatening complication associated with high rates of morbidity and mortality and requires prompt treatment. The majority of airway fistulae are iatrogenic complications of cancer treatments and present a particular challenge. There are different types of airway fistulae, including bronchopleural fistulae (BPF), tracheoesophageal fistulae (TEF), bronchoesophageal fistulae (BEF), gastrotracheal fistulae (GTF), and gastrobronchial fistulae (GBF) [1–5]. The main characteristic of airway fistulae is the atypical connection of the airway to the oesophagus, pleural space, and stomach.

The symptoms of airway fistulae are different and depend on the location of the fistula. Presenting symptoms of BPF can be cough, empyema, persistent air leak, and haemoptysis [5]. Significant dyspnoea can occur because of increased dead space ventilation if there is a massive air leak. Patients with TEF, BEF, GTF, or GBF can have varying clinical manifestations, depending on the rate of its formation, size, and location, and the comorbidities and immunological status of the patient. The main symptoms and signs are cough, aspiration, fever, dysphagia, pneumonia, haemoptysis, and chest pain^[1,4,6−9]^.

The therapeutic options for airway fistula include a range of surgical and medical techniques. The common approach is stenting to restore the integrity of the airway wall. The purpose of stenting is to palliate airway fistulae from symptoms of aspiration, dysphagia, worsening respiratory status, and poor nutritional status. In addition to stenting, the Amplatzer device is another minimally invasive choice for BPF treatment. The presence of two disks, one on each side of the defect, ensured good occlusion without impairing airway patency. The device induced local granulation that resulted in the total encapsulation of the occluder. Fruchter [5] and colleagues published their experience with the positioning of the Amplatzer atrial septal occluder in 10 patients, and the procedure had been well tolerated without side effects or complications. Furthermore, the Amplatzer atrial septal occluder has also been reported in TEF and BEF, with good results [14].

Currently, there is no consensus on the best choice of airway fistula treatment. Both covered metallic stents and the Amplatzer device are promising for minimally invasive airway treatment. Here, we report the experience of Amplatzer device application in airway fistula failure with a covered self-expandable metallic stent.

## Materials and methods

Informed consent was obtained from each patient. Ethics committee approval was obtained for this retrospective study. Patients who failed occlusion with a covered self-expandable metallic stent and received Amplatzer device (Shanghai Shape Memory Alloy Co., Ltd, China) placement from Jan 2015 to Jan 2022 were retrospectively enrolled. A total of 14 patients aged 42 to 66 years (55.14 ± 7.87) were enrolled in this study. All 14 patients with an airway fistula failed to be occluded with a covered metallic stent and received Amplatzer device placement. Among the 14 patients, 6 had BPF, 3 had TEF and 5 had GBF. The physical condition of the patients was assessed with the Karnofsky Performance Status (KPS) score before and after Amplatzer device placement. The details are listed in Table 1.

**Table 1:** Demographic and clinical characteristics

Gender/years	Previous disease	Types of fistulas	Types of stents	During time (day)	Fistula Size (mm)	Amplatzer (Wrist-LA disc -RA disc) mm	KPS before/ after
M/42	Esophageal hiatal hernia	GBF	Y-AS1	96	6	ASD (8-18-16)	60/70
M/56	Esophageal cancer	GBF	Y-AS1	90	5	ASD (6-16-14)	60/70
F/62	Esophageal cancer	GBF	Y-AS1	100	3	ASD (6-16-14)	70/80
M/65	Esophageal cancer	GBF	Y-AS1	116	5	ASD (6-16-14)	60/80
M/65	Esophageal cancer	GBF	Y-AS1	112	6	ASD (8-18-16)	60/80
F/50	Stroke	ETF	I-ES	324	3	ASD (6-16-14)	70/80
M/48	Wound	ETF	I-ES	215	6	ASD (8-18-16)	70/80
M/52	Esophageal cancer	ETF	I-ES	240	6	ASD (8-18-16)	60/70
M/58	Lung cancer	BPF	Y-AS2	104	3	VSD (4-8-8)	60/70
M/40	Lung cancer	BPF	Y-AS2	121	5	VSD (6-10-10)	60/80
M/54	Lung cancer	BPF	Y-AS2	118	5	VSD (6-10-10)	60/80
M/56	Lung cancer	BPF	Y-AS2	122	3	VSD (4-8-8)	60/70
M/58	Lung cancer	BPF	Y-AS2	121	5	VSD (6-10-10)	60/80
M/66	Lung cancer	BPF	Y-AS2	108	5	VSD (6-10-10)	60/70

I-ES: tube shaped esophageal stent; Y-AS1: Y-shaped covered airway stent; Y-AS1: Y-shaped covered airway stent with one dead end branch

### The procedure of ventricular septal defect (VSD) placement for BPF

**Fig. 1:**
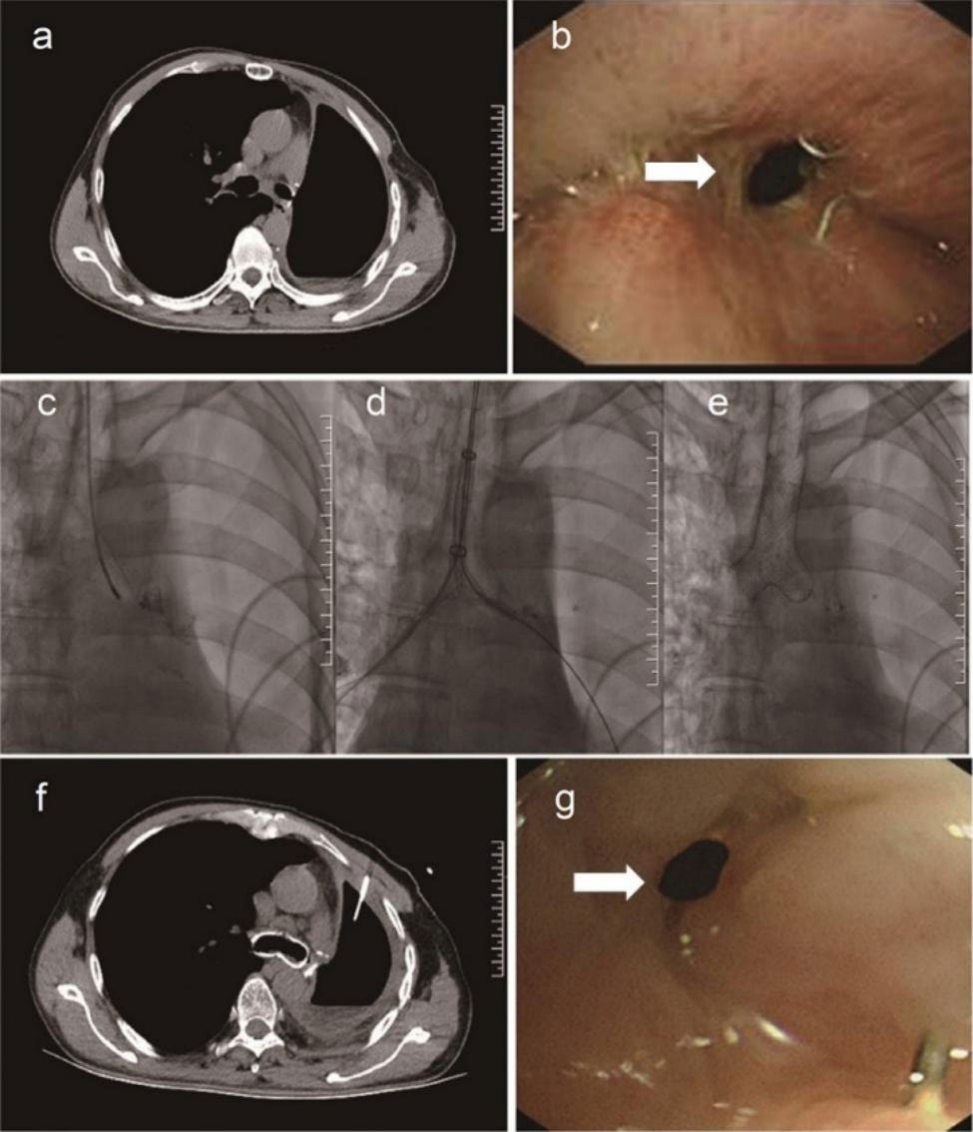
BPF patient failed to be cured by a Y-shaped covered metallic stent with one dead-end branch. CT (**a**) and bronchoscopy (**b**) showing the BPF (arrow) after pulmonary lobectomy. Bronchogram demonstrates the formation of BPF (**c**). The deployment of a Y-shaped covered metallic stent with one dead-end branch under the guidance of fluoroscopy (**d**). After stent placement, the BPF was occluded by the dead-end branch of the Y-shaped covered metallic stent (**e**). CT showed that the dead-end branch fit the residual bronchus (**f**). Three months post-stenting, the stent was removed due to granulation of the end of the stent, but the fistula (arrow) was not cured (**g**)

**Fig. 2:**
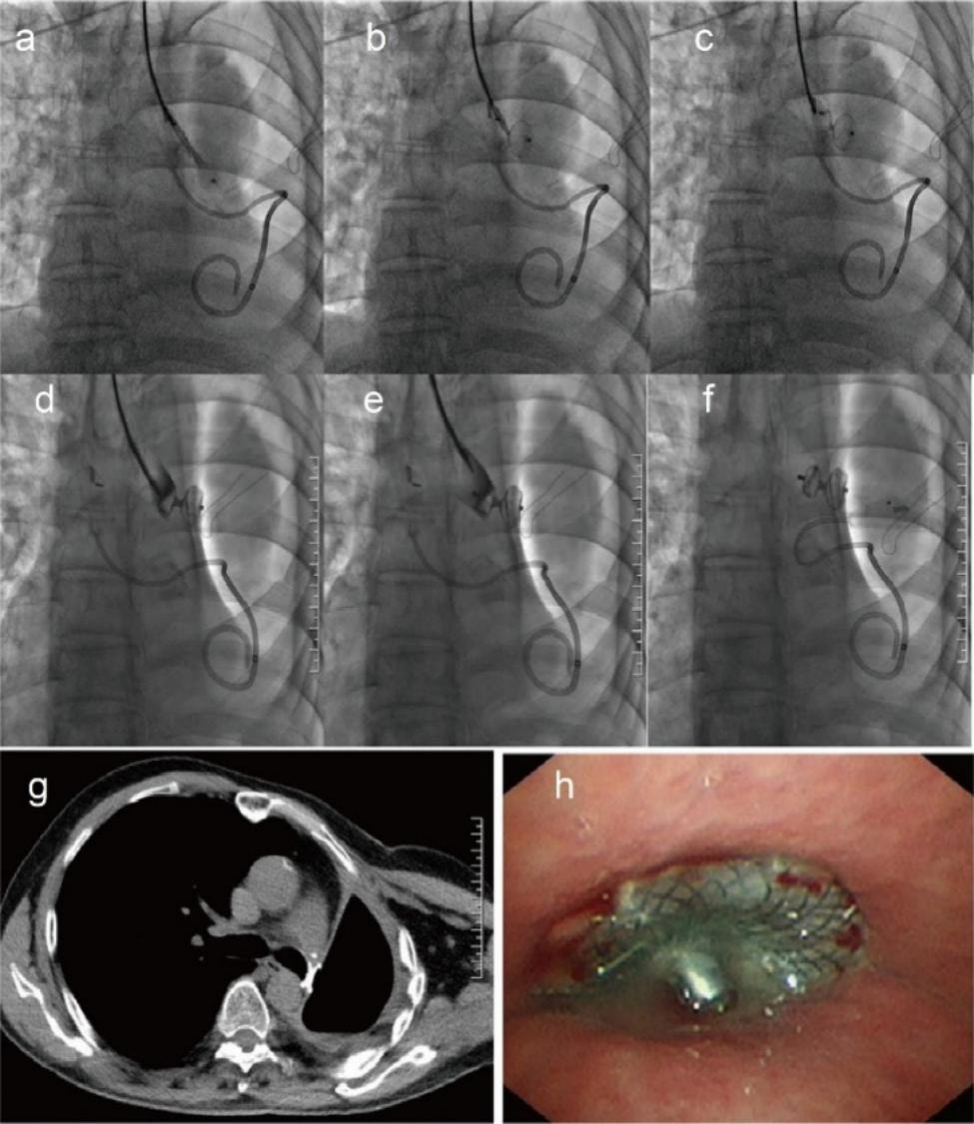
The process of VSD placement and follow-up. After the bronchogram, the sheath containing the VSD was introduced into the BPF. First, the distal disk was released (**a**) and pulled back to occlude the fistula (**b**). Then, the proximal disk was released to anchor the residual bronchus (**c**). The bronchogram via a catheter demonstrates the successful occlusion of BPF (**d**). The VSD was detached (**e**) and left in place to occlude the BPF (**f**). Representative images of VSD-occluded BPF from CT (**g**) and bronchoscopy (**h**)

After sedation, a guide wire with a catheter was introduced into the bronchus through the mouth under the guidance of fluoroscopy. Bronchography was performed by contrast agent injection via the catheter. After confirming the location of the BPF, a superstiff wire was introduced into the pleural space. Over the superstiff guide wire, a 5- to 7-F delivery catheter was introduced into the pleural space. The superstiff wire and catheter core were withdrawn. The VSD device was introduced through the BPF. The distal disk was released to oppose the fistula. The waist was extruded into the fistula. The proximal disk was placed in the residual bronchus. After the proximal disk was released, another bronchography was performed via a delivery catheter to confirm the successful occlusion of the BPF. Then, the device was detached (Figs. 1 and 2).

### The procedure of atrial septal defect (ASD) placement for TEF

**Fig. 3:**
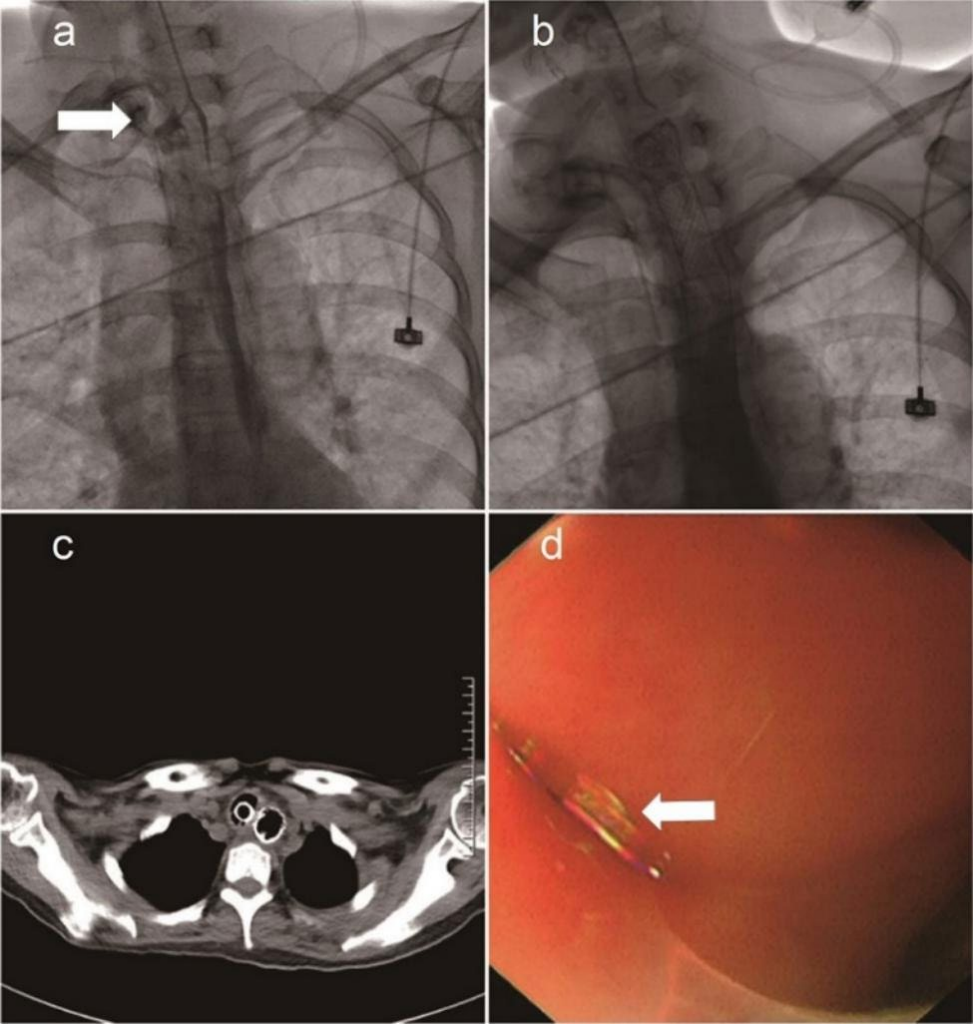
Oesophageal stent placement to treat ETF. The oesophagogram via a catheter demonstrates the communication (arrow) of the oesophagus and trachea (**a**). After stent placement, the fistula was occluded, and no contrast agent flowed into the trachea when the oesophagogram was performed via the catheter. CT also demonstrates successful occlusion (**c**). After the oesophageal stent was removed, a fistula (arrow) was observed by oesophagogastroscopy

**Fig. 4:**
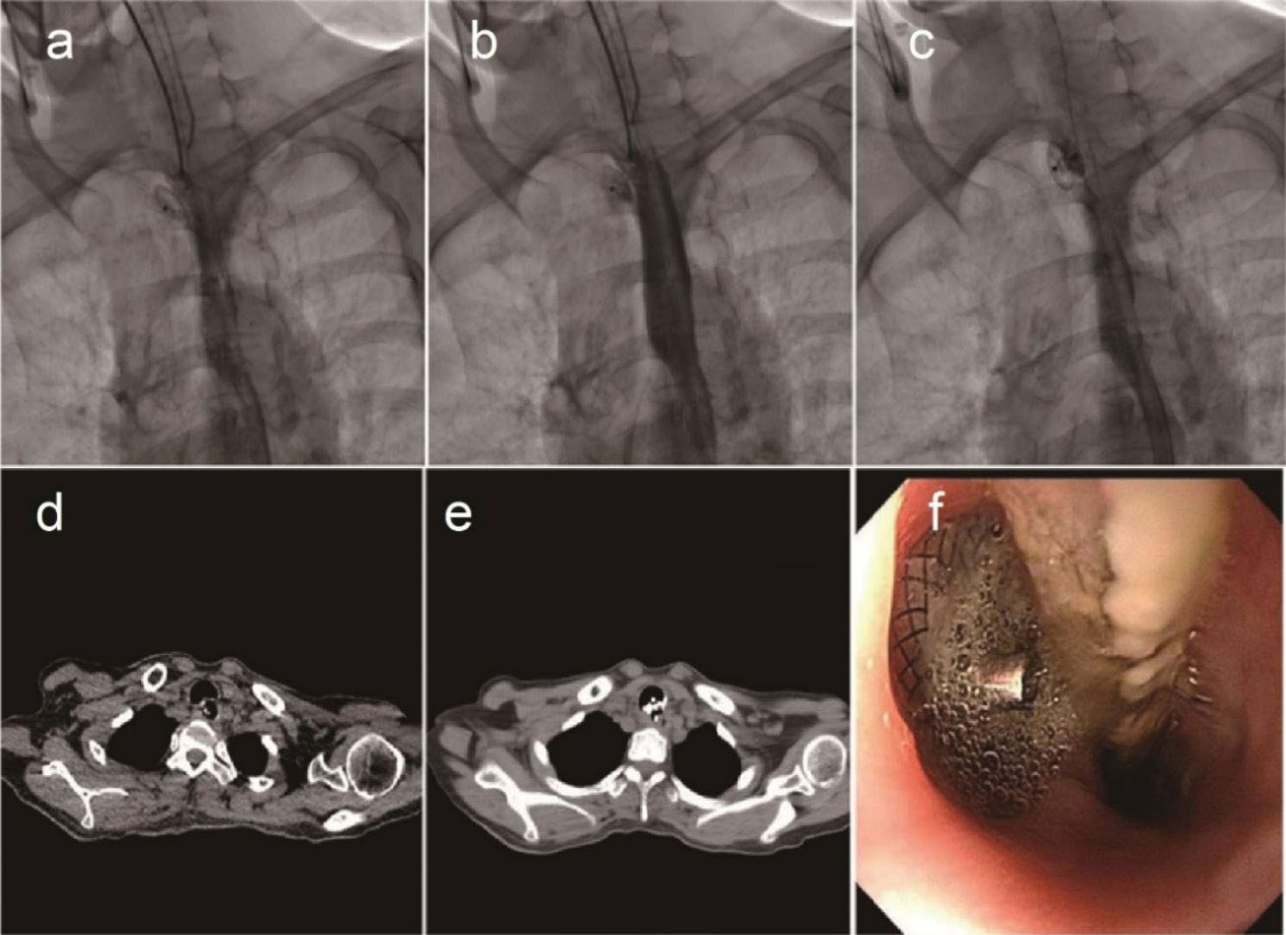
ASD placement to treat an ETF that failed to be cured by oesophageal stenting. The distal disk was released into the trachea (**a**). The proximal disk was released into the oesophagus. The oesophagogram via a catheter demonstrates the successful occlusion of the ETF (**b**). The ASD was detached and left in place to occlude the ETF (**c**). Representative CT image showing the fistula before (**d**) and after (**e**) ASD placement. Representative oesophagogastroscopy image of AD

After sedation, a guide wire with a catheter was introduced to the oesophagus through the mouth under the guidance of fluoroscopy. The catheter was adjusted, and the contrast agent was injected to confirm the fistula. The catheter and guide wire were introduced into the trachea, and a superstiff wire was exchanged. The tip of the super stiff guide wire was left in the trachea. Over the wire, a 5- to 7-F delivery catheter was introduced into the lumen of the trachea. The guidewire and catheter core were withdrawn. The ASD device was introduced through the TEF. The distal disk was released to oppose the tracheal membrane. The waist was extruded into the fistula. The proximal disk was placed into the wall of the oesophagus. After the proximal disk was released, the catheter was introduced to the near ASD device to perform oesophagography. After occlusion of the TEF was confirmed, the device was detached (Figs. 3 and 4).

### The procedure of ASD placement for GBF

**Fig. 5:**
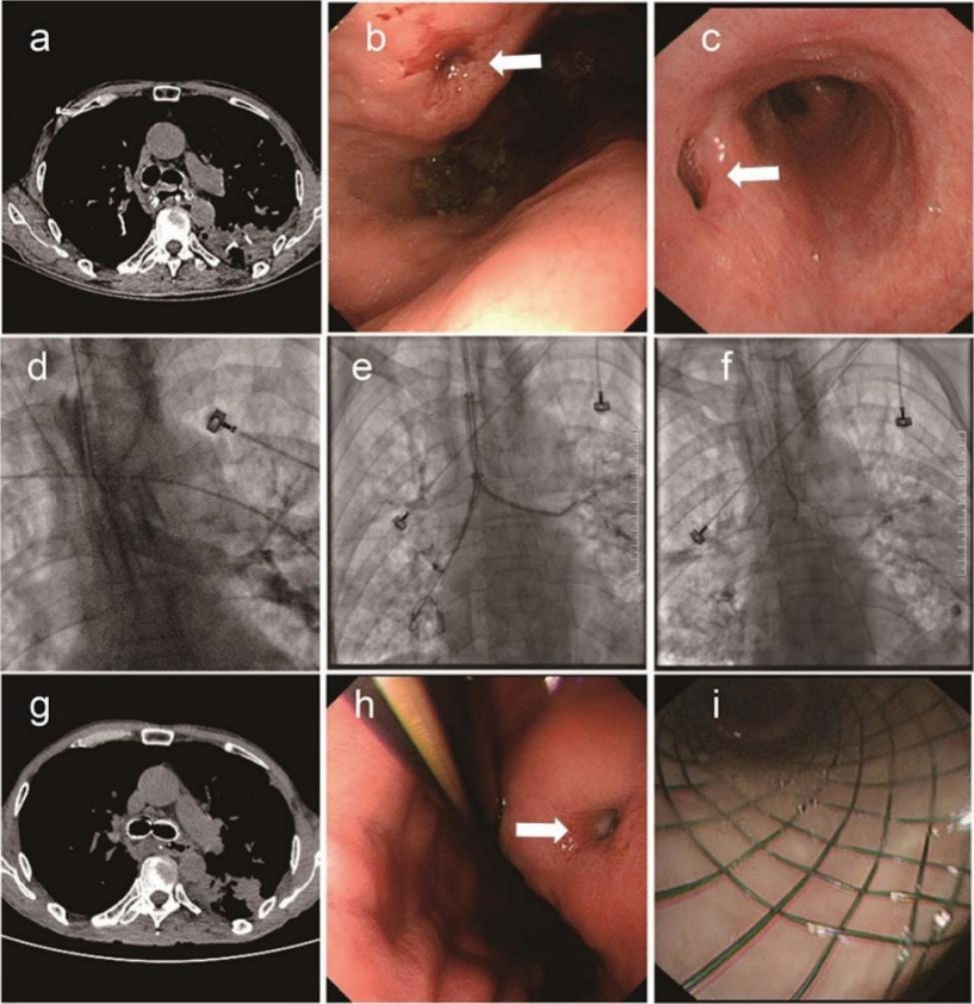
A GBF was treated by a covered Y-shaped self-expandable metallic stent. Representative images of GBF (arrow) from CT (**a**), gastroscopy (**b**), and bronchoscopy (**c**). Bronchogram was performed to find the GBF (**d**). Then, a covered Y-shaped self-expandable metallic stent was placed (**e**) and released (**f**). After stent placement, CT (**g**), gastroscopy (**h**), and bronchoscopy (**i**) were performed again to observe the GBF

**Fig. 6:**
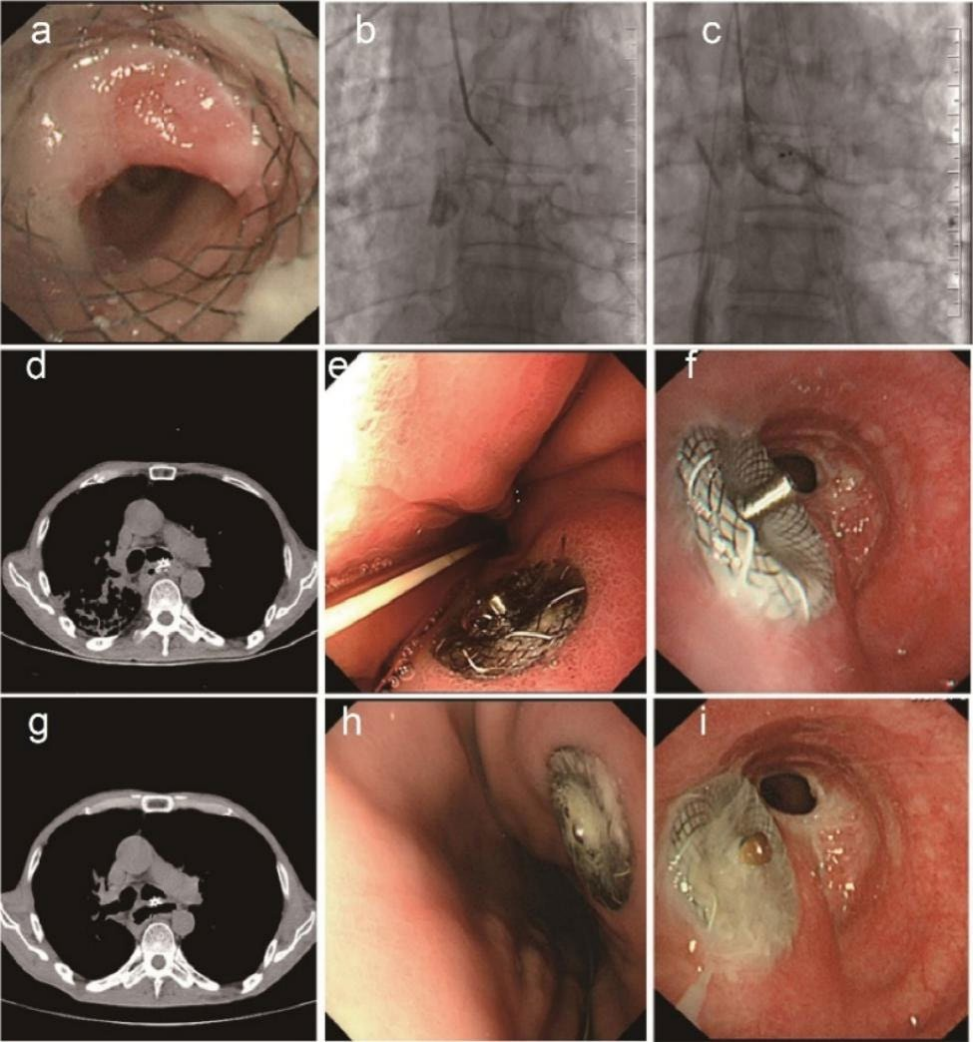
ASD placement to treat a GBF the failed to be cured by a covered Y-shaped self-expandable metallic stent. The covered Y-shaped self-expandable metallic stent was removed due to granulation hyperplasia (**a**). Bronchogram demonstrated that the GBF was not cured by the stent (**b**), and an ASD was placed to occlude the GBF (**c**). Representative images of ASD-occluded GBFs from CT (**d**), gastroscopy (**e**), and bronchoscopy (**f**). Representative images of ASD from CT (**g**), gastroscopy (**h**), and bronchoscopy (**i**) 6 months after placement

After sedation, a guide wire with a catheter was introduced into the bronchus through the mouth under the guidance of fluoroscopy. Bronchography was performed by contrast agent injection via the catheter. After confirming the location of the GBF, a super stiff wire was introduced into the stomach. Over the wire, a 5- to 7-F delivery catheter was introduced into the stomach. The guidewire and catheter core were withdrawn. The ASD was introduced through the GBF. The distal disk was released to oppose the gastric wall. The waist was extruded into the fistula, and the proximal disk was placed into the bronchus. After the proximal disk was released, another bronchogram was performed via the catheter to confirm the successful occlusion of the GBF, and the device was detached (Figs. 5 and 6).

After Amplatzer device placement, chest CT and bronchoscopy (Olympus, Japan) were performed 1 to 3 days after the procedure in patients with BPF. For patients with TEF and GBF, another oesophageal gastroscopy was performed. At 1, 3, and 6 months postprocedure, the patients were followed up.

### Statistical analysis

Descriptive statistics (mean and standard deviation) were used to summarize the data. Continuous variables were compared before and after Amplatzer device placement using Wilcoxon tests for paired samples. A p value less than 0.05 was considered statistically significant. Statistical analyses were performed using SPSS 22.0.

## Results

The average stent time was 141.93 ± 65.83 days. The size of the fistula ranged from 3 to 6 mm. All 14 Amplatzer occluders were successfully placed under the guidance of fluoroscopy. No procedure-related complications occurred. No contrast agent leakage was observed from the previous fistula. After Amplatzer device placement, the KPS score improved from 62.14 ± 4.26 to 75.71 ± 5.13 (P < 0.05).

During the 1-month, 3-month, and 6-month follow-ups, 13 of the Amplatzer occluders were surrounded with granulation, but none of them were totally embedded. No fistula recurrence was observed. VSD occlusion failed in only 1 patient due to the recurrence of lung cancer. Finally, he received a Y-shaped covered stent placement. The other 5 BPF patients with Amplatzer device placement recovered well in the 12-month follow-up. Among the 3 patients with ETF, the Amplatzer device was coughed up at the 7-month follow-up in one patient, and an oesophageal stent was placed to occlude the fistula. One patient was lost to follow-up, and 1 patient recovered well. Of the 5 patients with GBF, 2 died of tumour recurrence before finishing the 1-year follow-up. One patient received covered airway stent placement and Amplatzer device removal at the 8-month follow-up due to Amplatzer device migration. The other 2 patients were followed up for 1.5 years, and no migration was observed.

## Discussion

In the current study, we explored the outcomes of the Amplatzer device in treating airway fistula, including BPF, TEF, and GBF. All patients failed to have occlusion by a covered stent. The technique of Amplatzer device placement under the guidance of fluoroscopy alone is feasible. All of the Amplatzer devices in 14 patients successfully occluded the fistulae with no procedure-related complications. This presents an excellent short-term outcome.

Patients with airway fistula usually present with critical complications and are in poor clinical condition, which is accompanied by a high risk of anaesthesia and surgery. Minimally invasive treatment, including endoscopic therapy, transcutaneous therapy, and fluoroscopic therapy, has emerged as an effective option in recent years^[[10, 11]]^. The medical materials used in the minimally invasive treatment of airway fistula include glue, clips, coils, Watanabe spigots, silicone stents, and covered stents [12–15]. According to the size and location of the fistula, the strategies of fistula treatment are different. When a fistula is larger than 5 mm, a covered stent is the best choice [6, 7]. Other options are feasible in a small fistula less than 5 mm.

For stenting of BPF, Dutau [16] and colleagues reported their experience with a custom-made self-expandable covered metallic stent in 7 patients with large postpneumonectomy BPF. The air leak was stopped in all patients after stent placement, but two stent migrations were noted, which led to the failure of complete occlusion of the BPF. In a previous study, we reported the experience of different types of customized airway stents in treating BPF [7]. A total of 143 of 148 patients with BPF successfully received stenting at the first attempt, and 141 patients experienced symptom relief at the 30-day follow-up. However, there are still some cases of migration of stents in which there was failure to occlude the fistula and lead to deterioration of the general condition. For stenting of the TEF and BEF, Wang et al. [8]. reported 63 malignant airway fistulae treated with covered I-shaped, L-shaped, and Y-shaped stents. The mean survival time was 163 days (2-270 days). Most symptoms are relieved after stent insertion. The mean Karnofsky score increased from 43.0 ± 10.7 before stent placement to 66.7 ± 10.8 after stent insertion. However, incomplete closure and leakage were found in 18 patients. For stenting of the TGF and TBF, we used a Y-shaped covered metallic stent to occlude the fistula, and 59 cases (93.65%) were successfully implanted at the first attempt, with procedure times ranging from 5 to 10 min [6]. Oesophagograms with water-soluble iodinated contrast showed that the fistulae were completely covered with no contrast flowing into the airways and lungs and with the stents fully expanded. Four cases (6.35%) of incomplete or recurrent fistula closure were encountered.

Recently, the Amplatzer device emerged as a new option for the treatment of airway fistula and have obtained incredible results. Except for BPF reported in a small number of case series, the majority of ETF, GTF, and GBF studies were case reports [17–21]. In this study, all 14 patients failed to achieve stent occlusion. An Amplatzer device was adopted to occlude the fistula and obtain a good result. The Amplatzer devices were double-disk occluders, representing a large family of devices designed originally for transcatheter closure of cardiac septal defects or patent ductus arteriosus. The devices are made of a nitinol mesh with a central connector between the disks. In this study, we used the Amplatzer device to occlude airway fistulae that failed to occlude with airway stents or oesophageal stents. The Amplatzer device has a central waist with different diameters, and the distal and proximal disk diameters are 14 and 10 mm, respectively, and are larger than the diameter of the central waist. A polyester fabric inside the mesh facilitates occlusion and tissue growth over the device. The waist is placed inside the fistula, and the disks anchor the device on either side. In the treatment of BPF, there was one disk in the pleural space and one in the residual bronchus. In the treatment of TEF and GBF, there was one disk in the airway and one in the digestive tract.

Theoretically, the application of the Amplatzer device created a raw healing surface on the wall of the trachea or bronchus. The surrounding soft tissue will develop oedema, coaptation, and subsequent fibrosis of the applicated edges of the fistula site, leading to auto-obliteration. Although the process takes a long time, the Amplatzer device prevents the leakage of air and fluid. This improved the quality of life. In this study, we observed surrounding tissue hyperplasia embedded at the edge of the Amplatzer device after placement for 3 months and little granulation tissue hyperplasia at the 6-month follow-up. A totally embedded Amplatzer device was not observed in the 13 patients during the follow-up. The Amplatzer device dropped into the pleural cavity in one patient due to tumour recurrence at the 6-month follow-up. However, no massive bleeding occurred. Another airway stent was placed for palliative respiratory symptoms. The current data demonstrate the safety and efficiency of the application of the Amplatzer device in an airway fistula that failed to be occluded with a covered stent.

## Conclusion

In conclusion, the application of the Amplatzer device is a safe and effective option in the treatment of an airway fistula that failed to be occluded with a covered stent.

## Electronic supplementary material

Below is the link to the electronic supplementary material.

Supplementary Material 1

## Data Availability

Please contact the corresponding author for data requests.

## List of abbreviations

SEMS: Self-expandable metallic stent
CT: Computed tomography
VSD: Ventricular septal defect
ASD: Atrial septal defect
BPF: Bronchopleural fistula
TEF: Tracheoesophageal fistula
BEF: Bronchoesophageal fistula
GTF: Gastrotracheal fistula
GBF: gastrobronchial fistula
KPS: Karnofsky Performance Status
