# Supplementary material for: Covered SEMS failed to cure airway fistula closed by an amplatzer device
Source: BMC Pulm Med. 2023 Jul 20;23:270. doi: 10.1186/s12890-023-02548-8 (PMC10357874; doi:10.1186/s12890-023-02548-8)
Supplement: Supplementary file 1 — Supplementary Material 1 [file 12890_2023_2548_MOESM1_ESM.pdf]

Editing Certificate

This document certifies that the manuscript

Covered SEMS failed to cure airway fistula closed by an Amplatzer device

prepared by the authors

Huibin Lu; Yahua Li; Kewei Ren; Zongming Li; Juanfang Liu; Xuhua Duan; Jianzhuang Ren; Xinwei Han

was edited for proper English language, grammar, punctuation, spelling, and overall style by one or more of the highly qualified native English speaking editors at AJE.

This certificate was issued on **May 13, 2023** and may be verified on the AJE website using the verification code **E7DF-7DCD-EF81-ECBC-459E** .

Neither the research content nor the authors' intentions were altered in any way during the editing process. Documents receiving this certification should be English-ready for publication; however, the author has the ability to accept or reject our suggestions and changes. To verify the final AJE edited version, please visit our verification page at aje.com/certificate. If you have any questions or concerns about this edited document, please contact AJE at support@aje.com.

AJE provides a range of editing, translation, and manuscript services for researchers and publishers around the world. For more information about our company, services, and partner discounts, please visit aje.com.
